# A colorimetric assay to rapidly determine the activities of lytic polysaccharide monooxygenases

**DOI:** 10.1186/s13068-018-1211-z

**Published:** 2018-08-02

**Authors:** Damao Wang, Jing Li, Ann C. Y. Wong, Finn L. Aachmann, Yves S. Y. Hsieh

**Affiliations:** 1 Division of Glycoscience, Department of Chemistry, School of Engineering Sciences in Chemistry, Biotechnology and Health, KTH Royal Institute of Technology, AlbaNova University Center, 106 91 Stockholm, Sweden; 2 Wallenberg Wood Science Center, KTH Royal Institute of Technology, 100 44 Stockholm, Sweden; 3 Affinity Proteomics, SciLifeLab, School of Engineering Sciences in Chemistry, Biotechnology and Health, KTH Royal Institute of Technology, 171 21 Solna, Sweden; 4 Department of Physiology, Faculty of Medicine, School of Medical Sciences, The University of New South Wales, Sydney, NSW 2052 Australia; 5 Department of Biotechnology and Food Science, NTNU Norwegian University of Science and Technology, 7491 Trondheim, Norway

**Keywords:** Lytic polysaccharide monooxygenase, Enzyme assay, Biomass deconstruction

## Abstract

**Background:**

Lytic polysaccharide monooxygenase (LPMOs) are enzymes that catalyze the breakdown of polysaccharides in biomass and have excellent potential for biorefinery applications. However, their activities are relatively low, and methods to measure these activities are costly, tedious or often reflect only an apparent activity to the polysaccharide substrates. Here, we describe a new method we have developed that is simple to use to determine the activities of type-1 (C1-oxidizing) LPMOs. The method is based on quantifying the ionic binding of cations to carboxyl groups formed by the action of type-1 LPMOs on polysaccharides. It allows comparisons to be made of activities under different conditions.

**Results:**

Based on the colorimetric detection and quantification of the pyrocatechol violet (PV)-Ni^2+^ complex, we have developed an assay to reliably detect and quantify carboxylate moieties introduced by type-1 LPMOs. Conditions were optimized for determining the activities of specific LPMOs. Comparisons were made of the activities against cellulose and chitin of a novel AA10 LPMO and a recently reported family AA11 LPMO. Activities of both LPMOs were boosted by hydrogen peroxide in the 1st hour of the reaction, with a 16-fold increase for the family AA11 LPMO, and up to a 34-fold increase for the family AA10 LPMO.

**Conclusions:**

We developed a versatile colorimetric cation-based assay to determine the activities of type-1 LPMOs. The assay is quick, low cost and could be adapted for use in industrial biorefineries.

## Background

**Scheme 1:**
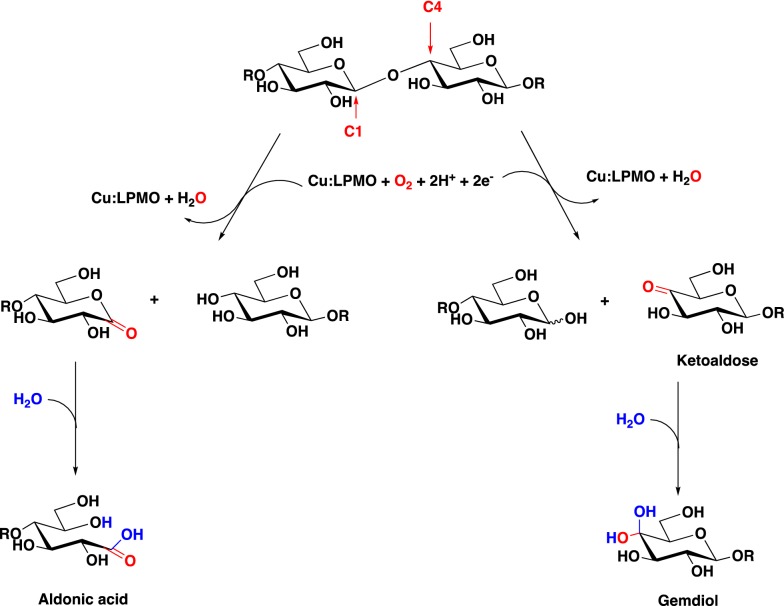
LPMOs catalyze oxidation within a polysaccharide chain leading to chain cleavage. Type-1 (C1) LPMOs oxidize at the C1 position resulting in the formation of a lactone, which is hydrated to generate an aldonic acid at the reducing end. Type-2 (C4) LPMOs oxidize at the C4 position, resulting in the formation of a ketoaldose at the non-reducing end (The figure is modified from Beeson et al. [5] and Hemsworth et al. [26])

Lytic polysaccharide monooxygenases (LPMOs) are enzymes that oxidatively cleave polysaccharides and provide auxiliary activity (AA) in the degradation of polysaccharides. They have a catalytic mono-copper center positioned on a flat enzyme surface that allows interaction with the polysaccharides and occur in a series of AA families [1]. LPMOs of the families AA9, AA10, AA11 and AA15 can oxidatively break glycosidic bonds on the surfaces of cellulose and chitin [2–4] (Scheme 1). This enzymatic activity has been thought to be dependent on the availability of O_2_ and a reducing agent [1, 5]. However, a recent report showed that LPMOs prefer H_2_O_2_ over O_2_ as a co-substrate and the report suggested that the low catalytic activities observed in previous studies were likely to be due to an absence of endogenous H_2_O_2_ [6]. This new finding that LPMO redox activity can be enhanced by H_2_O_2_ alone and the activity is proportional to the concentration could have a significant impact for their use in biorefineries [4].

LPMOs were first reported in 2010 [1]. Despite intensive research, measurement of their activities remains a problematic task largely hampered by the low solubility of the products and because cleaved oligosaccharides can be re-absorbed back on to the surface of insoluble polysaccharides. Indeed, conventional reducing sugar assays are unsuitable for the detection of the products. Although techniques such as X-ray photoelectron spectroscopy (XPS) can detect C(=O)OH functional groups on insoluble polysaccharides introduced by type-1 LPMO, it has a low throughput and limited quantitative precision. Other currently available assay methods for LPMOs require laborious procedures such as a post-treatment with polysaccharide hydrolases to convert the insoluble oxidized polysaccharides into small, soluble oligosaccharides each with a reducing-end aldonic acid. These oligosaccharides are then separated and quantified by high-performance anion-exchange chromatography with pulsed-amperometric detection (HPAEC-PAD) using a strong alkali eluent [7, 8]. Such methods are accurate but time-consuming and require a library of aldonic acid oligosaccharide standards derived from different polysaccharides, limiting the study of LPMO activity to specialized laboratories. Several other LPMO assays have also been reported, including one in which chitin was radiolabelled with ^14^C on C-2 and the quantity of soluble ^14^C oligosaccharides released by LPMO activity determined [9]. A microplate-based assay has also been developed that is based on labelling of reducing-end aldonic acid with a fluorescence dye [10]. The chromogenic substrate 2,6-dimethoxyphenol (2,6-DMP) has also recently been used to quantify H_2_O_2_ consumption by LMPOs. The LPMO catalyzes the oxidation of 2,6-DMP to form the chromogenic product coerulignone at the expense of two H_2_O_2_. However, the result did not correlate with the oxidative activity on the polysaccharide substrates [11]. A more convenient method of screening and comparing LPMO oxidative activities on different polysaccharide is urgently needed. Because oxidation at C1 by type-1 LPMOs generates aldonic acid at the reducing ends (Scheme 1), this contributes to the overall negative charge on the treated polysaccharide surface. This led us to develop an ion adsorption/desorption assay to quantify type-1 LPMO activities.

**Scheme 2:**
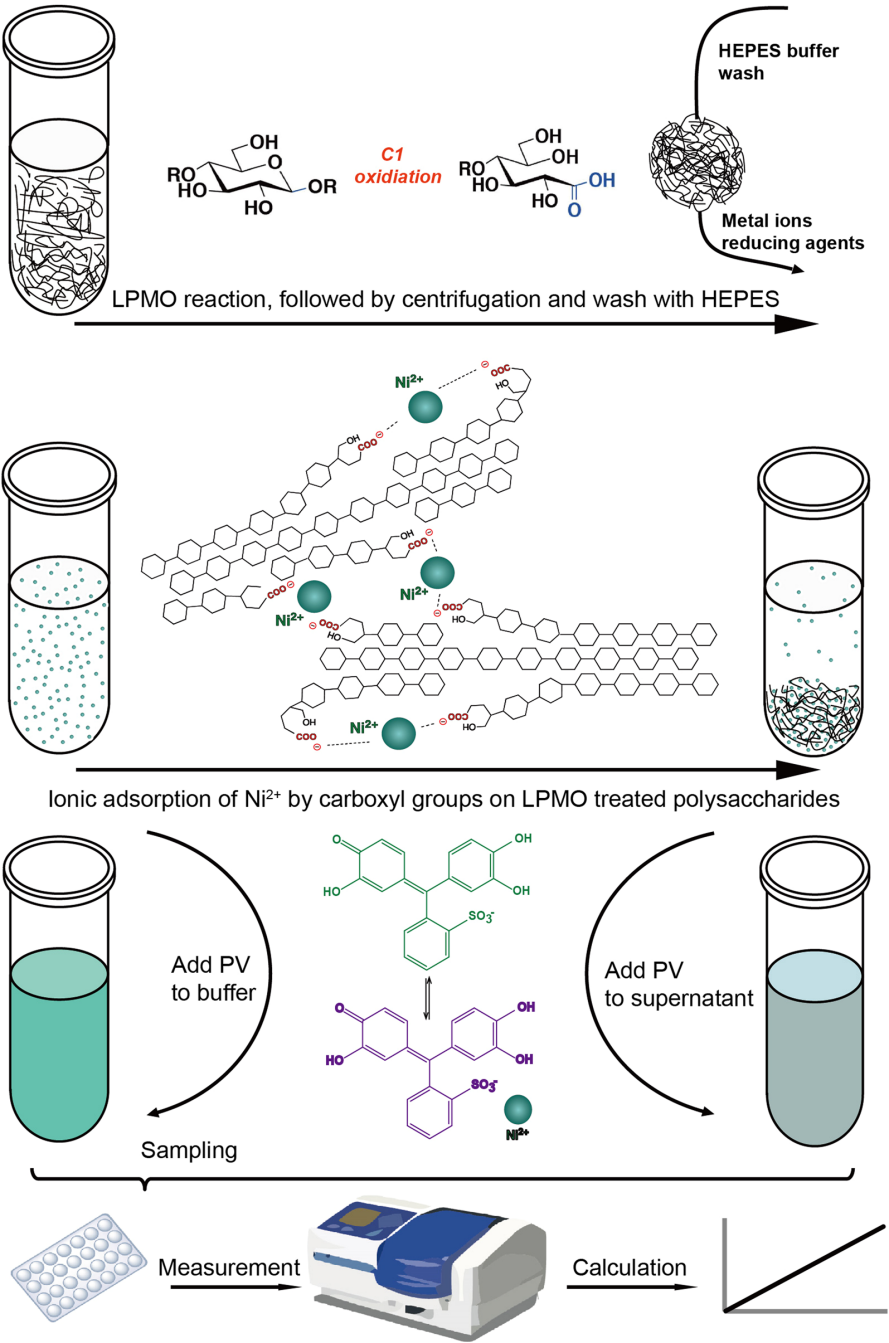
Schematic diagram of the colorimetric assay of type-1 (C1) LPMO enzyme activity. After LPMO treatment, the surface oxidized polysaccharide was precipitated by centrifugation, and soluble moieties including enzyme, metal ions and reducing agent were removed as supernatant. Polysaccharides were washed with HEPES buffer before Ni^2+^ was added to the polysaccharide surface carboxylates for ionic adsorption. A change in Ni^2+^ concentration in the supernatant was measured using pyrocatechol violet (PV) as an indicator. Absorbance was recorded and converted to numbers of carboxylate group introduced by the LPMO

There have been studies on quantifying charged groups on the surfaces of micro- and nano-particles using the adsorption and desorption of colored or fluorescent ionic dyes together with colorimetry or fluorimetry. However, the methods involved tedious washing steps and suffered badly from non-specific surface binding [12–15]. A method to quantify acrylic acid on the surfaces of insoluble polymethyl methacrylate (PMAA) microparticles without using a conventional ionic dye has also been developed [16]. In this study, it was demonstrated that Ni^2+^ efficiently bound to carboxylate moieties on the PMAA surface. Unbound Ni^2+^ in the supernatant was quantified spectrophotometrically using the complexometric indicator pyrocatechol violet (PV). Inspired by the simplicity and convenience of this method, we further developed it into a quick screening method for type-1 LPMO activity and for the relative quantification of carboxylate moieties that have been grafted onto the surface of recalcitrant polysaccharides (Scheme 2). The method maybe applicable for optimizing the use of LPMOs in biomass biorefineries.

## Results and discussion

### Optimization of the stability of the Ni^2+^-pyrocatechol violet (PV) complex

**Fig. 1:**
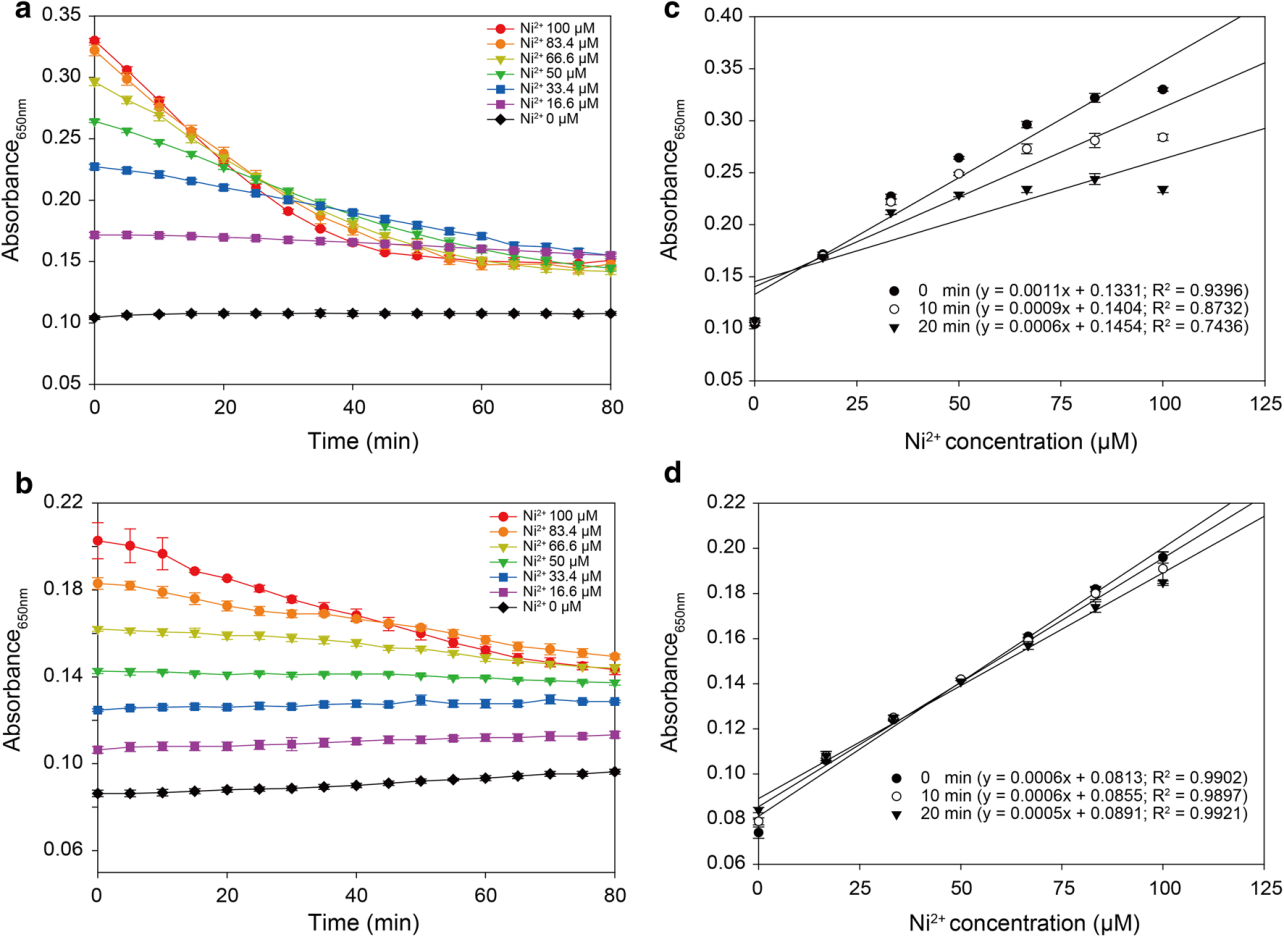
Stability of Ni^2+^–PV complex in 10 mM HEPES (pH 8) (**a**), and in the ethanol/HEPES mix (95:5 v/v) (**b**). Correlation curves of absorbance/Ni^2+^ concentrations after 0, 10 and 20 min incubation with PV, in 10 mM HEPES (pH 8) (**c**), and in the ethanol/HEPES mix (95:5 v/v) (**d**). Error bars represent SD from a total of three independent measurements

Pyrocatechol violet (PV) is a classic complexometric indicator that chelates transition metal cations including Cu^2+^, Ni^2+^ and Cd^2+^. The formation of the metal-PV complex changes the solution absorption spectrum from medium turquoise to bluish-violet in color and is unaffected by other buffer additives such as 100 mM Na^+^, Mg^2+^ or Ca^2+^[17]. Previous research indicated that of the various transition metal cations tested, Ni^2+^ had the highest color response and sensitivity [16]. However, the Ni^2+^-PV complex itself is not stable. In our study, when PV was added to Ni^2+^ in the slightly alkaline buffer (HEPES buffer pH 8) used by Hennig et al. [16], the color of the Ni^2+^-PV complex faded quickly at a higher concentration of Ni^2+^ (50 μM), after 20 min incubation, there was over a 30% decrease in the absorbance at 650 nm, suggesting a low stability constant between Ni^2+^ and PV under the conditions described (Fig. 1a). We found that the ethanol:10 mM HEPES buffer (pH 8) (95:5 v/v), instead of simply the 10 mM HEPES buffer (pH 8), could stabilize the complex, resulting in a smaller decrease in absorbance at 650 nm (Fig. 1b). Although using this mixture narrowed the range of absorbance variation, the linearity remained stable (*R*^2^ > 0.99 after 20 min). Coefficient of variation of absorbance with the mixture was 2.88, much lower than the 16.98 with the HEPES buffer (Fig. 1c, d). Furthermore, the ethanol:10 mM HEPES buffer (pH 8) (95:5 v/v) can precipitate water-soluble polysaccharides including CM cellulose, facilitating the separation of unbound Ni^2+^ and Ni^2+^ bound to the CM-cellulose (Scheme 1). Therefore, the amount of Ni^2+^ surface bound to the negatively charged groups, which in this case are carboxylate groups, can be calculated by the simple subtraction of unbound Ni^2+^ from the total Ni^2+^.

**Fig. 2:**
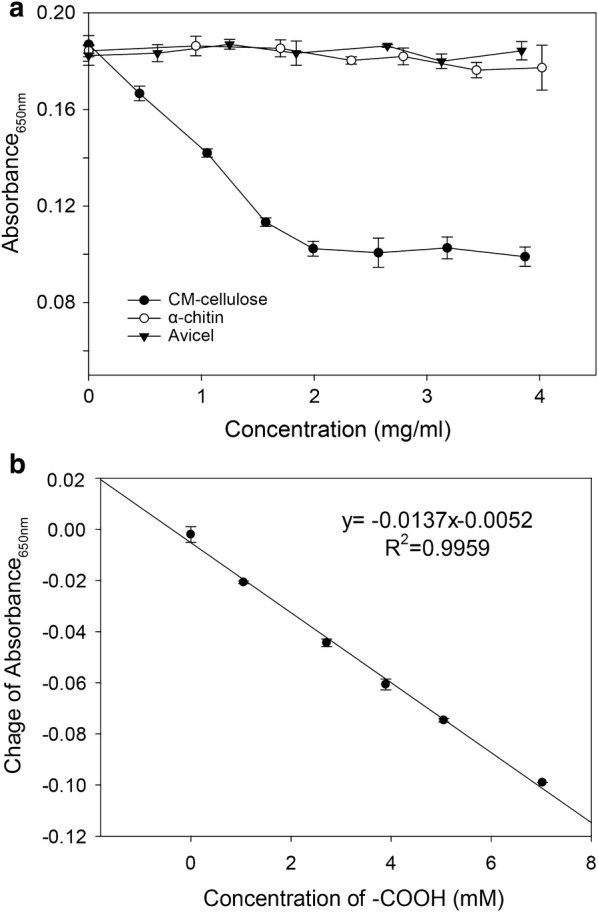
Monitoring of Ni^2+^ (2 mM) binding to three different substrates, CM-cellulose, α-chitin and Avicel (**a**), and correlation of Ni^2+^ cations reduced by carboxyl groups of CM-cellulose (50% substitution), the changes of absorbance were obtained by the values as read minus the background at 0 mM of carboxyl groups (**b**). Error bars represent SD from a total of three independent measurements

Based on this method and using the conditions we had optimized, we tested the three polysaccharides CM cellulose, α-chitin and Avicel for Ni^2+^ cation adsorption. Absorbances at 650 nm of the Ni^2+^–PV complex were measured at different polysaccharides concentration (Fig. 2a). The results indicated that the uncharged neutral polysaccharides α-chitin and Avicel showed, respectively, either no ionic adsorption of Ni^2+^ cations or only very weak adsorption. We thus excluded the possibility that Ni^2+^ cations adsorb to neutral polysaccharides. By contrast, the CM cellulose has negative charged carboxyl group that can absorb Ni^2+^ cations. The observed ratio of Ni^2+^ cations:carboxyl groups was not the theoretical 1:2, possibly due to not all the carboxyl groups on the CM cellulose being accessible for binding. The concentration of Ni^2+^ cations in the supernatant was strictly in inverse proportion to the concentration of carboxylate moieties on the CM-cellulose surface (Fig. 2a) and was linearly proportional (*R*^2^ = 0.9959) (Fig. 2b). With this in mind, we applied the method to quantify the carboxylate moieties generated by the recently reported *Fusarium fujikuroi* AA11 LPMO (*Ff*AA11) [18].

### Detection of LPMO AA11 activity using the cation-based assay

**Fig. 3:**
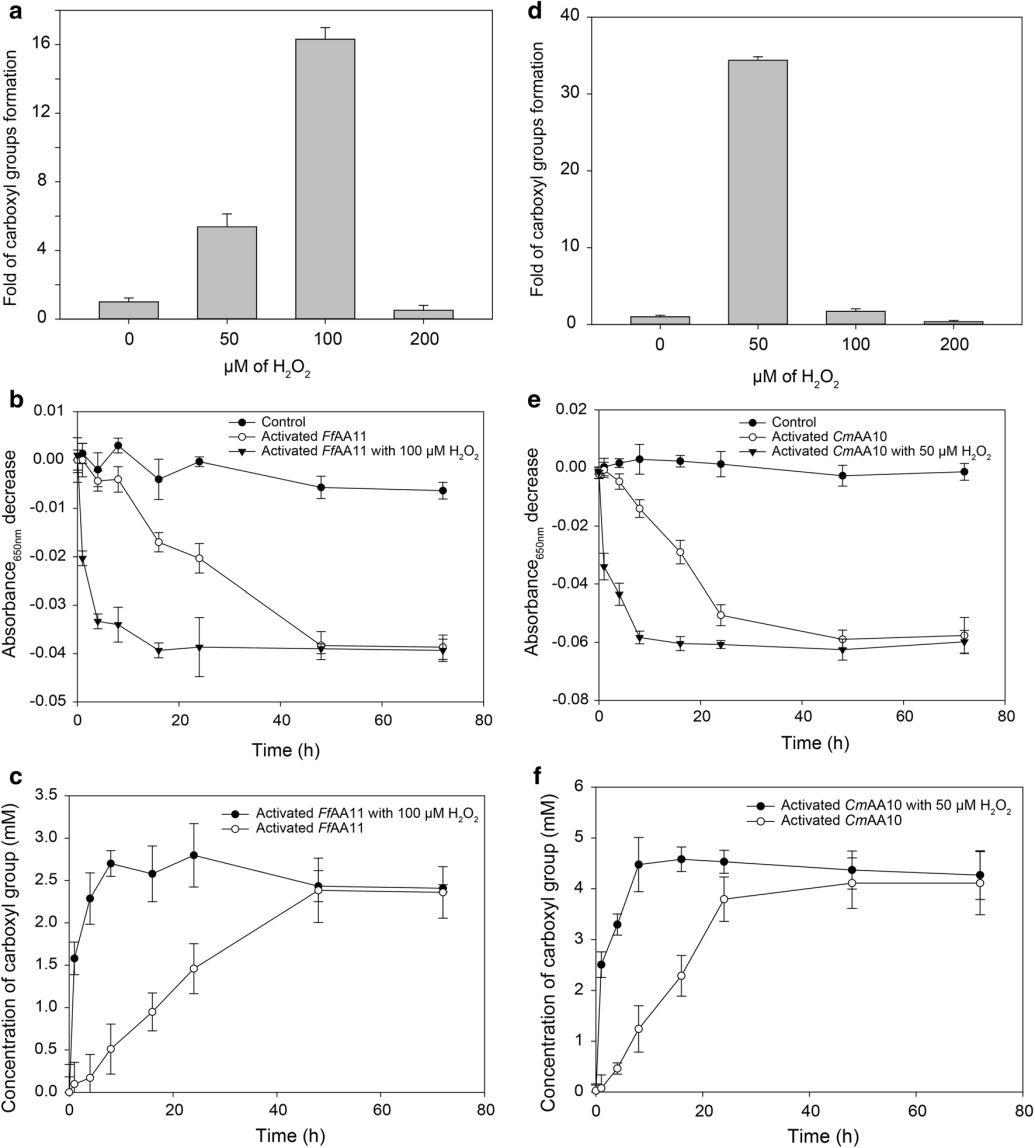
The optimal H_2_O_2_ concentration for the production of carboxylate moieties by *Ff*AA11 (**a**) and *Cm*AA10 (**b**) was calculated in folds compared with the reaction in the absence of H_2_O_2_. Ni^2+^ adsorption and the formation of total carboxylate groups over time in (1) *Ff*AA11-treated α-chitin, with or without 100 µM H_2_O_2_ (**c**, **d**), and in (2) *Cm*AA10-treated Avicel with or without 50 µM H_2_O_2_ (**e**, **f**). Error bars represent SD from total of three independent measurements

Activities of the LPMOs in the AA11 family have not been well documented [3, 18]. Using highly crystalline α-chitin from shrimp shells as substrate, together with O_2_ and ascorbic acid as the reducing agent for the redox reaction, our assay showed *Ff*AA11 to have a *V*_max_ of 0.06 mM/h, and a TN (turnover number) of 0.5 min^−1^, which is in agreement with other studies of particular LPMOs that are considered not to be catalytically efficient enzymes [19]. However, given that H_2_O_2_ may be the preferred co-substrate as suggested by recent literature [6, 9], we examined the activity of this LPMO with and without H_2_O_2_ as the co-substrate. By titration using different concentrations of H_2_O_2_, our assay showed the optimal condition to be 100 µM H_2_O_2_ for 1 h, leading to an almost 16-fold increase in product formation compared with the reaction without H_2_O_2_ (Fig. 3a). The calculated *V*_max_ was 0.79 mM/h and the TN 6.6 min^−1^. Our study clearly showed that using 100 µM H_2_O_2_ for 1 h boosted *Ff*AA11 catalytic activity (Fig. 3b, c), whereas it was about 5.4-fold and 0.5-fold for 50 and 200 μM H_2_O_2_, respectively. Thus, the assay permits simple comparisons of LPMO activity, providing better biological insights, and has encouraged us to exploit the assay further on uncharacterized LPMOs.

### Detection of the activity of a novel LPMO AA10 using the cation-based assay

**Fig. 4:**
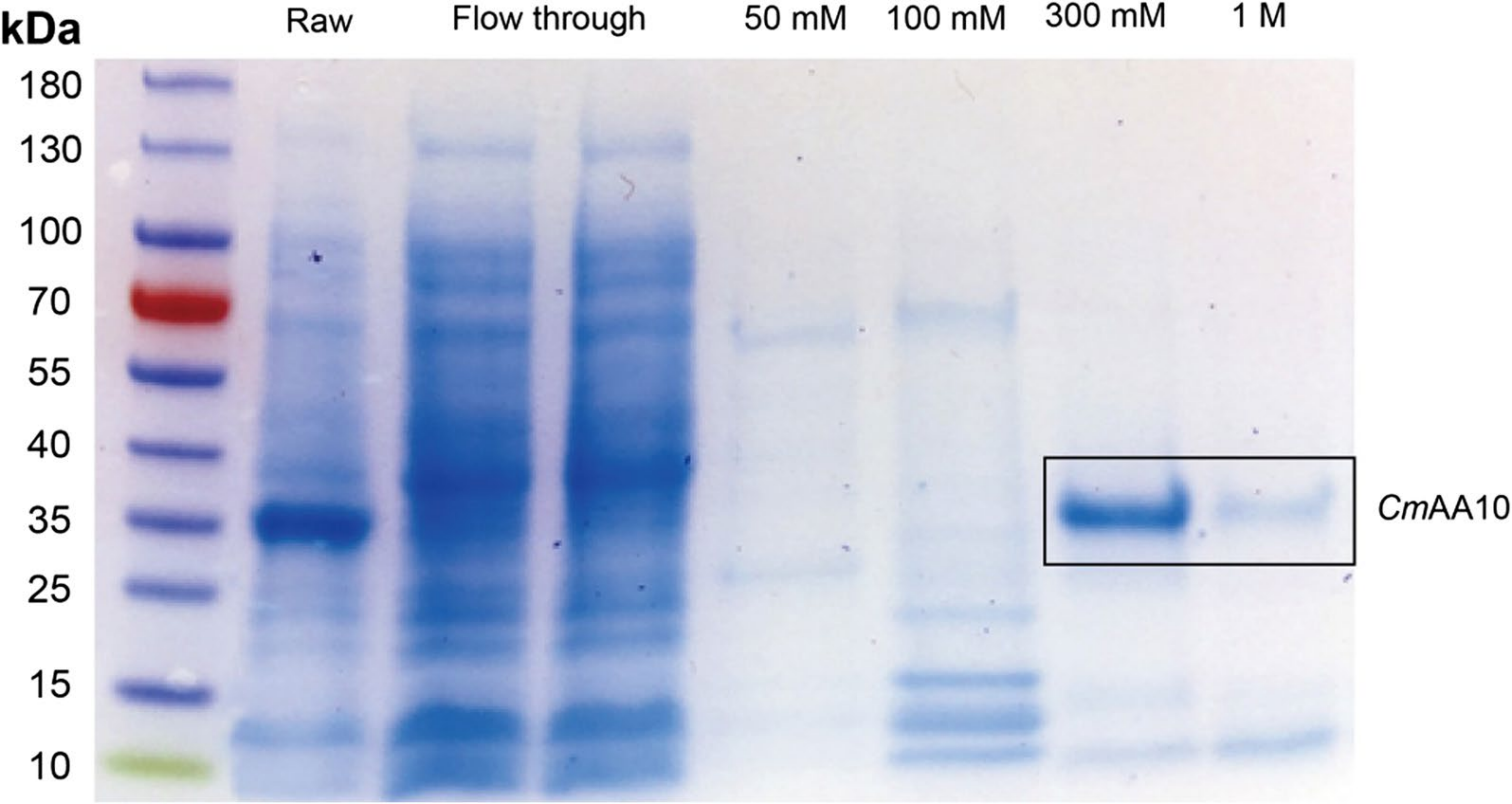
SDS-PAGE of *Cm*AA10 purification, including raw protein from *E. coli* periplasm, flow through and fractions from elution by different concentrations of imidazole

**Fig. 5:**
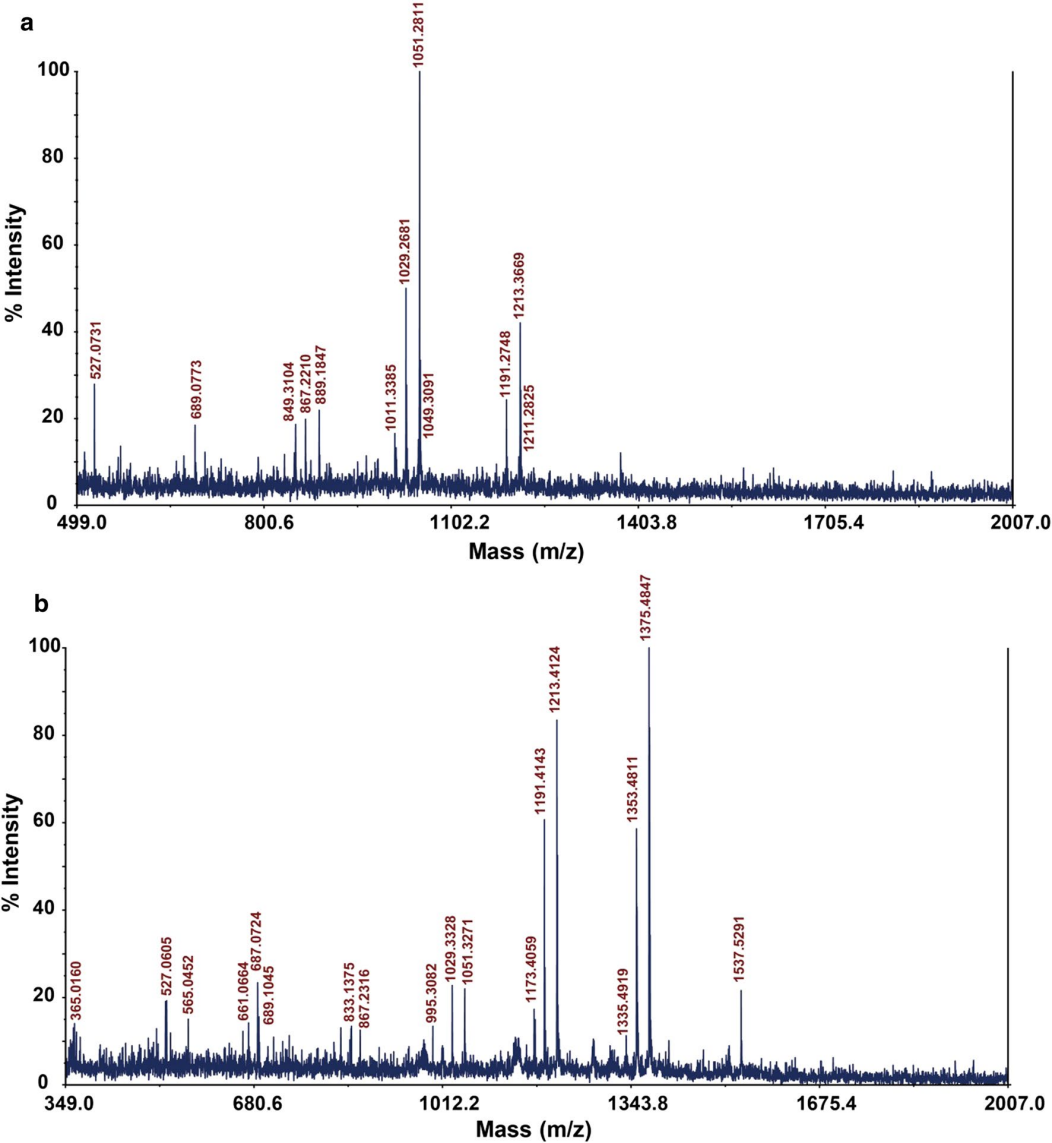
MALDI-TOF/TOF MS analysis of the reaction products of CmAA10 treated **a** Avicel and **b** PASC. DP2 + Na^+^ (365.02), DP3 + Na^+^ (527.06), DP3_al_^−^ + 2Na^+^ (565.05), DP4/DP4_-2_ + Na^+^ (689.1/687.07), DP5_al_ + Na^+^ (867.23), DP6_al_ + Na^+^ (1029.33), DP6_al_^−^ + 2Na^+^ (1051.33), DP7_-2_ + Na^+^ (1173.41), DP7_al_ + Na^+^ (1191.41), DP7_al_^−^ + 2Na^+^ (1213.41), DP8_-2_ + Na^+^ (1335.49), DP8_al_ + Na^+^ (1353.48), DP8_al_^−^ + 2Na^+^ (1375.48), DP9_al_^−^ + 2Na^+^ (1537.53). Masses of dehydrated oligosaccharides formed by phosphoric acid treatment are 661.07, 833.14 and 995.31 for DP3, DP4 and DP5, respectively

To detect activity in an uncharacterized, putative LPMO, we cloned the putative chitin-binding protein *Cm*AA10 (Genbank ID: WP_039915231.1) from the bacterium *Cellvibrio mixtus*. The protein was produced heterologously in *E. coli* and was purified to homogeneity (Fig. 4). Interestingly, we found the protein showed type-1 LPMO activity not against α-chitin but strictly against the crystalline cellulose Avicel and the amorphous cellulose phosphoric acid swollen cellulose (PASC) (Fig. 5). We used the assay with Avicel as the polysaccharide substrate to screen the activity of the protein at different H_2_O_2_ concentrations. Unlike *Ff*AA11, we found that the optimal co-substrate concentration for *Cm*AA10 was 50 µM H_2_O_2_ (Fig. 3d), with the activity being abolished at 100 µM H_2_O_2_. As has been shown for AA9 and AA10, there is a fine balance between conditions where the LPMOs are catalytically active and stable and where they are self-oxidizing [6, 9, 20, 21]. Being a more potent LPMO, the reaction with *Cm*AA10 was complete within 8 h, whereas catalysis without H_2_O_2_ continued over 48 h before reaching a plateau (Fig. 3e, f), suggesting reaction rate can be regulated by H_2_O_2_ alone. Indeed, at the optimal H_2_O_2_ concentration, *Cm*AA10 has a *V*_max_ of 4.1 mM/h and a calculated TN of 68.3 min^−1^. A 34-fold increase in catalytic activity was achieved in 1 h of enzyme action compared with the treatment without H_2_O_2_ (*V*_max_ = 0.12 mM/h; TN = 2 min^−1^). Altogether, the activity studies on *Cm*AA10 and *Ff*AA11 are in good agreement, as both display the highest LPMO activity against cellulose and chitin with H_2_O_2_ as the co-substrate. Furthermore, H_2_O_2_ did not interfere with polysaccharide substrate binding.

### Concluding remarks

For comparing the concentrations of carboxylate moieties introduced by type-1 LPMOs under different conditions, this Ni^2+^ cation-based assay was found to be rapid and reliable. We demonstrated the Ni^2+^ cation-based assay is useful for measuring LPMO activity; multiple measurements can be achieved in 1 h on a 96-well plate. Compared with other reported assays, no post-enzymatic treatment, radioactive tracer or chemical labelling is needed. The cation-based assay has the potential to become a general method for comparing the activities of proteins within other LPMO families, such as the starch-specific AA13 family [22] or the xylan-specific AA14 family [23], or for investigating LPMO proteins within families with polysaccharide substrate specificity that are waiting to be discovered.

There are also limitations to this Ni^2+^ cation-based assay. First, we observed a discrepancy in the stoichiometric amounts of reductant and product that is most likely due to the CM cellulose standards we used for the carboxylate/Ni^2+^ calibration curve, or to the presence of other reductants from unexpected sources. Second, the Ni^2+^-carboxylate complex does not follow a 1:2 ratio as the spacing between the carboxylate moieties introduced by LPMO cannot be precisely regulated. For this reason, a better standard to simulate the aldonic acid introduced by LPMOs would certainly increase the reliability of the method. Finally, as this Ni^2+^ cation-based method does not detect C4-oxidized products generated by type-2 LPMOs, but it offers a way of distinguishing between LPMOs with type-1 and 2 activities.

In summary, a simple and stable colorimetric assay has been developed to investigate type-1 LPMO activity. This easily adaptable and scalable assay has the potential to be developed into a fully automated screening assay for LPMO oxidative activities against different polysaccharide substrates, including cellulose and chitin, and also water-soluble polysaccharides that can be precipitated by 95% ethanol, such as heteroxylans and xyloglucans. Finally, the assay can be scaled up for different applications in both academic and industrial settings that involve the synergistic use of polysaccharide hydrolases and LPMOs, which would allow their used to be further exploited and optimized.

## Methods

### Colorimetric measurements on the Ni^2+^–PV complex in different buffer solutions

NiCl_2_ solutions in 10 mM HEPES buffer (pH 8) or in ethanol:HEPES buffer (95:5 v/v) at 0–100 µM concentrations (500 µL each) were prepared. Each concentration was mixed with an equal volume of 400 µM pyrocatechol violet solution (PV, 500 µL) freshly made in 10 mM HEPES buffer or in ethanol:HEPES before use. The solutions were vigorously mixed and their absorbance at 650 nm was recorded immediately and every 5 min for 80 min in total. Correlation curves and equations were obtained by linear fitting. Other buffers were also tested, but we found the ethanol:HEPES buffer (95:5 v/v) provided the most stable Ni^2+^-carboxylate complex.

### Colorimetric measurement of the reduction in concentration of the Ni^2+^ cation caused by ionic adsorption to carboxyl groups

Carboxymethyl (CM) cellulose with a known degree of substitution and molecular weight (Megazyme, Wicklow, Ireland; DS = 0.5; DP = 1000) was used as a polysaccharide standard. The amount of carboxylate was calculated based on the equation:

$$\upmu {\text{mole}}\;{\text{of}}\;{-}{\text{COOH}} = \frac{{{\text{Mass}}\;{\text{of}}\; {\text{CM}} - {\text{cellulose}}\;\left( {\text{mg}} \right)}}{{{\text{Mw}}\;{\text{of}}\;{{\text{C}}_6}{{\text{H}}_7}{{\text{O}}_2}{{\left( {\text{OH}} \right)}_x}{{\left( {{\text{OC}}{{\text{H}}_2}{\text{COOH}}} \right)}_y}}} \times 50\% \times 1000$$

*y *= 0.5 (degree of substitution); *x*  +  *y *= 3; *x*  =  3 − *y* (degree of substitution).

In an optimized procedure, CM-cellulose (or AA10 and AA11 treated polysaccharides) was added to 540 µL ethanol-HEPES buffer (ethanol:10 mM HEPES, pH 8 = 95:5, v/v) giving CM-cellulose concentrations of 1.05, 2.71, 3.89, 5.05 and 7.03 mM. The solutions were mixed well and left undisturbed for 2 min. NiCl_2_ in ethanol:HEPES buffer (2 mM, 60 µL) was then added to the CM-cellulose solutions. After vigorous mixing, the solutions were left at ambient temperature for 2 min, and then centrifuged (16,000×*g*, 5 min) to precipitate Ni^2+^–CM cellulose particles. The supernatant (500 µL) was removed and mixed vigorously with PV (500 µL, 80 µM), giving a final PV concentration of 40 µM. The absorbance was recorded immediately. A standard curve was plotted from CM cellulose with a known degree of substitution (DS) using absorbance and concentration of carboxyl group (in the initial 600 µL = 540 µL of buffer + 60 µL of 2 mM NiCl_2_) derived from the above equation. Non-specific absorption of Ni^2+^ to Avicel (Sigma, MO, USA) and α-chitin (Sigma, MO, USA) was also examined using the procedure described above.

### Preparation of *Ff*AA11 and novel *Cm*LPMOAA10

Fungal chitin-specific *Ff*AA11 was prepared as described by Wang et al. [18]. The sequence (c.73-c.1026) of putative chitin-binding protein (WP_039915213), namely *Cm*AA10, was chemically synthesized by GeneArt (ThermoFisher Scientific, MA, USA). To produce the mature protein with catalytic *N*-terminal His-1 on the expressed protein, the *Cm*AA10 gene was ligated to the pelB leader sequence using overlap PCR. The chimera cDNA sequence (pelB-*Cm*AA10) was amplified by PCR using Q5 HF polymerase master mix (New England Biolabs, MA, USA) and subsequently cloned into the pET-26b(+) vector (Invitrogen) between *Nde*I and *Xho*I restriction sites. The sequence was verified by EMBL (Heidelberg, Germany). The primers and gene sequences (codon optimized) are shown in Table 1. The final constructs were transformed into *Escherichia coli* BL21 star (DE3) cells by heat shock treatment at 42 °C for 45 s, and the cells were grown and selected on LB plates + kanamycin (50 mg/L). Transformant *E. coli* cells harboring the *Cm*AA10 were grown in LB broth (BD) containing 50 mg/L kanamycin, at 37 °C on an orbital shaker (200 rpm) until the absorbance at 600 nm reached 0.6‒0.8. Protein production was induced by 0.5 mM isopropyl β-d-1-thiogalactopyranoside (IPTG) (Amresco, OH, USA) using the optimized temperature of 16 °C and shaking at 180 rpm for 18 h; the cells were harvested by centrifugation (4000×*g*, 15 min). The cell pellets were resuspended in 30 mM Tris–HCl (pH 8) buffer containing 1 mM EDTA and 20% (w/v) sucrose at a ratio of 1:50 (wet cell weight: volume in mL). The mixture was gently shaken at room temperature for 10 min, and the cells recovered by centrifugation (16,000×*g*, 30 min at 4 °C). The pellets were rapidly resuspended in ice-cold water, gently shaken for 10 min and centrifuged (16,000×*g*, 30 min). The supernatant containing periplasmic proteins was harvested, passed through an affinity HisTrap column (GE Healthcare), and the target protein eluted with a gradient of increasing imidazole concentration. The purified recombinant protein was concentrated using an Amicon ultracentrifugal filter unit (molecular weight cut-off value of 10,000 Da, Millipore), and the concentration was determined using the Bradford assay (Bio-Rad, CA, USA).

**Table 1:** Primers used for cloning of *CmAA10*

Primer	Sequence (5′–3′)
pelB leader forward	CATATGAAATACCTGCTGCC
pelB-*CmAA10* reverse	CAAAGCCATGGGCCATCGCC
pelB-*CmAA10* forward	GGCGATGGCCCATGGCTTTG
*CmAA10* reverse	CTCGAGACGCGGTGCATTAC

The purified LPMO was saturated with copper by incubation with a threefold molar excess of CuCl_2_ for 1 h at 30 °C. Excess copper was removed by desalting the protein using a PD MidiTrap G-25 desalting column (GE Healthcare). The reactions were set up with 1% (w/v) cellulose substrates including the crystalline cellulose preparation Avicel (Sigma, MO, USA), and the amorphous cellulose PASC prepared as described by Zhang et al. [24]. The substrates were incubated in Cu^2+^ saturated *Cm*AA10 in 1 mM ascorbate, and 50 mM sodium phosphate buffer (pH 6.0). After 16 h incubation at 37 °C, the mixture was centrifuged and the supernatant collected for further analysis.

Analyses of enzymatic reaction products were performed by MALDI‒TOF MS (Applied Biosystems, CA, USA). The reaction product (5 µL) was mixed with 10 mM NaCl (3 µL) and 2,5-dihydroxybenzoic acid (10 mg/mL, 5 µL) in 50% (v/v) acetonitrile [25]. Then 1 µL of the mixture was spotted onto a stainless steel plate and rapidly dried under vacuum for homogeneous crystallization. The spectrometry was done using an accelerating voltage of 20,000 V with a delay time of 200 ns. The spectrometer was operated in the reflection mode.

### Study of the effect of H_2_O_2_ on AA10 and AA11 activities using the Ni^2+^ cation ionic adsorption assay

To study the effect of H_2_O_2_, multiple reactions were set up with 1% (w/v) α-chitin as substrate and 2 µM of *Ff*AA11 (in 50 mM ammonium acetate, pH 5.0, 1 mM ascorbic acid), with H_2_O_2_ (0, 50, 100, 200 µM). The oxidized α-chitin was centrifuged (4000×*g*, 5 min), and the precipitate washed 6 times with 1 mL HEPES buffer. The washes were to remove any soluble enzymes and Cu^2+^ ions that could affect Ni^2+^ binding. The soluble aldonic acids were also removed in the washing steps, and only the carboxylate moieties left behind on the insoluble recalcitrant polysaccharides were analyzed.

The amounts of carboxylate moieties introduced were then quantified by the colorimetric assay described above. For *Cm*AA10 and Avicel, the reactions were set up with 1 µM enzyme, followed by the same assay for *Ff*AA11 as described above. Controls were set up by incubating the substrate with copper-free enzyme (without the pre-incubation of the enzyme with the threefold molar excess of CuCl_2_), and without ascorbic acid and H_2_O_2_. The maximum reaction velocities (*V*_max_) and turnover numbers (TN) of the LPMOs were calculated from nonlinear regression plots using Origin 9.0 software (OriginLab, Northampton, MA, USA).
